# Factors associated with nutritional status improvement in hospitalized oldest-old adults at nutritional risk

**DOI:** 10.3389/fnut.2026.1856364

**Published:** 2026-07-20

**Authors:** Shan Jiang, Xiling Chen, Lan Ma, Qihao Guo, Yuehui Wang, Xuan Qu, Yun Fan, Songbai Zheng, Xiaohong Liu

**Affiliations:** 1 Department of Geriatrics, Peking Union Medical College Hospital, Chinese Academy of Medical Sciences & Peking Union Medical College, Beijing, China; 2 Department of Geriatrics, The Second Affiliated Hospital of Zhengzhou University, Zhengzhou, Henan, China; 3 Department of Geriatrics, The Second Affiliated Hospital of Harbin Medical University, Harbin, Heilongjiang, China; 4 Department of Geriatrics, Shanghai Sixth People’s Hospital, Shanghai Jiao Tong University School of Medicine, Shanghai, China; 5 Department of Geriatrics, The First Hospital of Jilin University, Changchun, Jilin, China; 6 Department of Geriatrics, Beijing Hospital, Beijing, China; 7 Department of Geriatrics, Huadong Hospital Affiliated to Fudan University, Shanghai, China

**Keywords:** clinical outcomes, energy intake adequacy, geriatric nutrition, hospitalized older adults, malnutrition, Mini Nutritional Assessment–Short Form, nutritional intervention adherence, nutritional risk screening

## Abstract

**Introduction:**

Malnutrition is highly prevalent among hospitalized older adults and is associated with adverse clinical outcomes. However, the determinants of nutritional improvement and clinical implications of such improvement in oldest-old hospitalized patients remain insufficiently characterized. This study aimed to identify factors associated with nutritional improvement and to evaluate the relationship between this improvement and clinical outcomes among hospitalized patients aged ≥80 years who were at nutritional risk.

**Methods:**

This multicenter prospective cohort study included hospitalized patients aged ≥ 80 years at nutritional risk (Nutritional Risk Screening 2002 score ≥ 3) from geriatric wards of tertiary hospitals in China. Nutritional status was assessed using the Mini Nutritional Assessment–Short Form (MNA-SF) at admission and at 90-day follow-up. Nutritional improvement was defined as an improvement in MNA-SF category at 90 days. Multivariable logistic regression analyses were performed to identify factors associated with nutritional improvement and to examine the association between nutritional improvement and adverse clinical outcomes, including mortality, readmission, falls, and infection.

**Results:**

Among 675 patients included in the final analysis (mean age 88.5 ± 4.8 years; 63.7% male), 251 (37.2%) achieved nutritional improvement at 90 days. In multivariable analyses, a higher Charlson Comorbidity Index was associated with a lower likelihood of nutritional improvement (odds ratio [OR] = 0.751, 95% confidence interval [CI]: 0.665–0.849, *p* < 0.001). Achieving energy adequacy (OR = 2.424, 95% CI: 1.511–3.887, *p* < 0.001) and good adherence to nutritional interventions (OR = 1.921, 95% CI: 1.303–2.834, *p* < 0.001) were independently associated with nutritional improvement, whereas protein adequacy was not. Nutritional improvement was independently associated with reduced risks of readmission (OR = 0.545, 95% CI: 0.351–0.847, *p* = 0.007) and new infection (OR = 0.388, 95% CI: 0.225–0.671, *p* < 0.001) but was not significantly associated with mortality or falls.

**Conclusion:**

Among hospitalized oldest-old patients at nutritional risk, achieving adequate energy intake and maintaining adherence to nutritional interventions were independently associated with nutritional improvement. Nutritional improvement was associated with lower risks of readmission and infection. These findings highlight the potential clinical relevance of sustained nutritional monitoring and management in oldest-old hospitalized populations.

## 1 Introduction

Population aging has led to a substantial increase in the number of older adults requiring hospitalization, particularly those aged ≥ 80 years, a group often referred to as the oldest-old. Malnutrition and the risk of malnutrition are highly prevalent among hospitalized older patients and are associated with adverse clinical outcomes, including prolonged length of stay, functional decline, infection, readmission, and mortality (1–3). In the oldest-old population, these risks are further exacerbated by reduced physiological reserve and a higher burden of multimorbidity. Despite increasing recognition of its clinical importance, nutritional management in geriatric inpatients remains suboptimal, particularly in China, where recent large-scale data have shown insufficient implementation of nutritional interventions among hospitalized older adults (4). The determinants of meaningful nutritional improvement during hospitalization are not fully understood.

The Mini Nutritional Assessment–Short Form (MNA-SF) is widely used to evaluate nutritional status in older adults and has been validated as a reliable and practical screening tool for identifying malnutrition and the risk of malnutrition (5). It has also demonstrated prognostic value in predicting morbidity and mortality (6, 7). However, most existing studies have focused on baseline nutritional status as a predictor of outcomes rather than on dynamic changes in nutritional status over time (8). Whether improvement in nutritional status during and after hospitalization translates into better short-term clinical outcomes therefore remains unclear. Moreover, although clinical guidelines emphasize the importance of adequate energy and protein intake (9), real-world evidence identifying nutritional factors that independently contribute to nutritional recovery in the oldest-old hospitalized populations remains limited.

Achieving recommended energy and protein intake is often challenging in frail older adults because of acute illness, reduced appetite, multimorbidity, and functional impairment (10). These challenges are even more pronounced in the oldest-old population. Importantly, a discrepancy between energy and protein intake—often referred to as a “protein–energy gap”—has been commonly observed in hospitalized patients, where overall food intake is insufficient and dietary composition may not meet both energy and protein requirements (11). Adherence to prescribed nutritional interventions may therefore play an important role in achieving adequate intake (12). Nevertheless, few multicenter studies have examined the relative contributions of energy adequacy, protein adequacy, and adherence to nutritional interventions to subsequent improvements in nutritional status, particularly among patients aged ≥ 80 years. In addition, the relationship between nutritional improvement and clinically relevant outcomes, such as readmission, infection, falls, and mortality, remains poorly understood.

To address these gaps, we conducted a multicenter cohort study including hospitalized oldest-old adults (aged ≥ 80 years) who were at nutritional risk from 28 tertiary hospitals. The objectives of this study were to (1) identify factors associated with improvement in nutritional status at 90 days and (2) evaluate whether nutritional improvement was associated with reduced risks of adverse clinical outcomes, including readmission, infection, mortality, and falls. We hypothesized that adequate energy intake and better adherence to nutritional interventions would be independently associated with nutritional improvement and that such improvement would, in turn, be associated with reduced risks of adverse clinical outcomes.

## 2 Materials and methods

### 2.1 Study design and participants

This study was a predefined subgroup analysis of a registered prospective multicenter observational cohort (ClinicalTrials.gov identifier: NCT04751032). The parent registry enrolled hospitalized geriatric patients aged ≥ 65 years who were identified as being at nutritional risk. Findings from this cohort have been previously reported, including our prior study evaluating the effects of nutritional support in geriatric inpatients classified according to the Global Leadership Initiative on Malnutrition criteria (13). In contrast to the previous analysis, which focused on nutritional status classification and the effectiveness of nutritional support, the present study specifically targeted the oldest-old population (aged ≥ 80 years) and examined dynamic changes in nutritional status, particularly nutritional improvement, and their associations with clinical outcomes. For the present analysis, we restricted the study population to patients aged ≥ 80 years to examine nutritional improvement and its association with clinical outcomes in this high-risk subgroup. All other inclusion and exclusion criteria were consistent with the original study protocol.

The parent study was designed and coordinated by the Department of Geriatrics at Peking Union Medical College Hospital. Participants were recruited from geriatric wards of 28 tertiary hospitals across multiple provinces and municipalities in China. Nutritional risk was assessed at admission using the validated Nutritional Risk Screening 2002 (NRS 2002) tool (14). Eligible patients were consecutively enrolled between September 2020 and December 2022. The inclusion criteria for the present analysis were (1) age ≥ 80 years; (2) nutritional risk defined as an NRS 2002 score of ≥ 3; and (3) hospital stay ≥ 2 days. The exclusion criteria included (1) terminal illness with an expected survival time of ≤ 3 months; (2) transfer to another department during hospitalization; (3) contraindications to nutritional intervention (e.g., hemodynamic instability); and (4) participation in other nutrition-related research within the previous 6 months.

The study was conducted in accordance with the principles of the Declaration of Helsinki. The protocol was approved by the Ethics Committee of Peking Union Medical College Hospital (Approval No. ZS-2429; approved on August 20, 2020), and written informed consent was obtained from all participants or their legal representatives before enrollment.

### 2.2 Study procedures

Before study initiation, the principal investigators provided standardized training to participating geriatric physicians regarding the study protocol, implementation procedures, and study timeline. All participating physicians completed a standardized 6-h training program on in-hospital and post-discharge nutritional support before the start of patient enrollment. The training curriculum was primarily based on international clinical nutrition guidelines, particularly those from the European Society for Clinical Nutrition and Metabolism (ESPEN) (9, 15). Nutritional assessments and data collection procedures were conducted according to predefined study protocols to ensure consistency across participating centers.

At admission, hospitalized patients were screened for nutritional risk using the NRS 2002. The recommended daily energy intake was 25–30 kcal/kg body weight, calculated based on actual body weight unless clinically inappropriate (e.g., severe obesity), in which case adjusted body weight was used, and the recommended protein intake was 1.0–1.2 g/kg body weight. The minimum nutritional target was defined as achieving ≥ 75% of the recommended energy and protein requirements because this threshold has been suggested to reflect clinically meaningful adequacy of intake and has been used in previous clinical studies to indicate sufficient nutritional support (16).

However, because this was a real-world observational study, specific nutritional interventions were individualized according to patient clinical conditions and local clinical practice patterns and therefore were not fully standardized across centers.

Each participating center’s geriatric care team included a trained dietitian responsible for assessing patients’ daily energy and protein intake and assisting geriatric physicians in developing individualized nutritional support plans. Dietitians responsible for dietary assessment were not involved in outcome adjudication and were blinded to clinical outcomes during follow-up. Nutritional intervention followed a stepwise approach. First, patient usual diets were optimized to energy-dense fortified diets according to individual preferences and oral nutritional supplements (ONSs) were prescribed between meals. If dietary intake combined with ONSs provided < 50% of the target nutritional requirements within 5 days, escalation to enteral tube feeding or parenteral nutrition was recommended as clinically indicated. This stepwise approach was implemented as a guideline-based framework rather than a strictly mandatory protocol, allowing clinicians to individualize nutritional strategies according to patient clinical conditions.

During hospitalization, trained dietitians reassessed nutritional intake weekly based on dietary records, which were collected using a 24-h dietary recall method combined with nursing-assisted intake documentation. At discharge, geriatric physicians developed individualized outpatient follow-up and post-discharge nutritional plans. After discharge, nutritional counseling was provided every 2–4 weeks through outpatient visits or telephone follow-up and ONS prescriptions were issued when necessary. At 90 days after admission, patients attended a face-to-face outpatient visit for reassessment of nutritional intake and nutritional status. Patients who continued to take ONSs as prescribed at the 90-day follow-up were considered to have good adherence to the nutritional intervention, which was assessed based on patient self-report supplemented by prescription records and follow-up documentation.

### 2.3 Data collection and clinical outcomes

A dedicated database was established for the Chinese Nutritional Risk Screening and Intervention Registration Study to systematically collect demographic, clinical, nutritional, and follow-up data. Demographic variables included sex, age, height, body weight, and body mass index (BMI). Clinical data included acute diagnoses and pre-existing comorbidities, from which the Charlson Comorbidity Index (CCI) was calculated (17). Nutritional status was assessed using dietary intake records and the MNA-SF. The MNA-SF was administered within 48 h of admission and repeated at the 90-day follow-up to evaluate changes in nutritional status over time. Parameters of comprehensive geriatric assessment included the Barthel Index of Activities of Daily Living (ADL), handgrip strength, and calf circumference. Calf circumference was measured using a non-stretchable tape at the widest part of the calf with the patient in a supine or seated position. Treatment-related information included the nutritional treatment pathway, daily dietary intake, and the use of ONSs.

Handgrip strength was measured using a calibrated electronic dynamometer (Camry EH101, Guangdong, China). Participants performed two maximal 3-s contractions with each hand at intervals of ≥ 30 s, and the highest value was used for analysis. Muscle mass was estimated using calf circumference, measured in the seated position with the knee and ankle flexed at 90°, and the mean of the maximal circumferences of both calves was recorded. Functional status was assessed using the Barthel ADL Index (range 0–100), with higher scores indicating greater functional independence (18).

The primary clinical outcomes were adverse events occurring within 90 days after admission, including all-cause mortality, readmission, new infection, and falls. New infection was defined as an infection diagnosed by a certified medical institution after discharge that required antibiotic treatment, as documented in medical records or diagnostic reports. This included respiratory, urinary tract, and gastrointestinal infections supported by laboratory evidence such as elevated white blood cell counts or increased high-sensitivity C-reactive protein levels. Falls were self-reported during follow-up, and additional information regarding the cause of the fall and occurrence of fracture was recorded.

### 2.4 Statistical analyses

All statistical analyses were performed using SPSS version 26.0 (IBM Corp., Armonk, NY, USA). Normality of continuous variables was assessed using the Shapiro–Wilk test. Normally distributed data are presented as the mean ± standard deviation and non-normally distributed data as the median (interquartile range). Between-group comparisons were performed using the independent samples *t*-test or Mann–Whitney U test, as appropriate, and categorical variables were compared using the chi-square test or Fisher’s exact test. Patients were categorized into nutritional improvement and non-improvement groups based on changes in MNA-SF grade at 90 days. Multivariable logistic regression was used to identify factors associated with nutritional improvement. Variable selection was based on clinical relevance, prior literature, and univariable analysis (*p* < 0.10). Multicollinearity was assessed using variance inflation factors (VIFs), with all VIFs being <5. Separate logistic regression models were constructed to evaluate the association between nutritional improvement and 90-day clinical outcomes (mortality, readmission, falls, and new infection), adjusting for age, baseline BMI, CCI, and ADL score. Given the multicenter design, hospital-level clustering was not explicitly accounted for in the regression models, as the primary focus was on patient-level associations and to maintain model parsimony. Missing data were minimal (<5%) and were handled using complete-case analysis. A *post hoc* power analysis indicated that the sample size (*n* = 675) provided adequate statistical power (>80%). All tests were two-sided, and *p* < 0.05 was considered statistically significant.

## 3 Results

**Figure 1:**
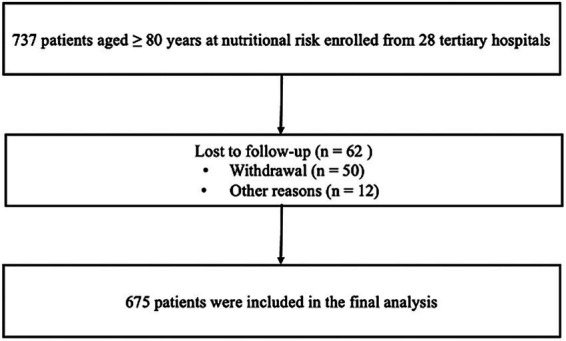
Flowchart of participant selection and inclusion.

A total of 737 hospitalized patients aged ≥ 80 years at nutritional risk were enrolled from the geriatric wards of 28 tertiary hospitals. Sixty-two patients did not complete the follow-up, and 675 were included in the final analysis (Figure 1). Comparisons between patients included in the final analysis and those lost to follow-up are presented in Supplementary Table S1. No clinically meaningful differences in baseline demographic or clinical characteristics were observed between the two groups. The mean age of the cohort was 88.5 ± 4.8 (range: 80–105) years, and 63.7% were men. The most common admission diagnoses included infection, stroke, coronary heart disease, malignancy, and heart failure. The cohort also had a high burden of comorbidities, particularly diabetes, hypertension, cardiovascular disease, and cerebrovascular disease. According to baseline MNA-SF classification, 330 patients were malnourished, 281 at risk of malnutrition, and 64 had normal nutritional status. At the 90-day follow-up, the distribution shifted to 169 malnourished patients, 334 at risk of malnutrition, and 172 with normal nutritional status. Based on changes in MNA-SF grade at 90 days, 251 (37.2%) patients were categorized into the nutritional improvement group (from “malnourished” to “at risk,” or from “at risk” to “normal”) and 424 (62.8%) into the non-improvement group. When stratified by baseline nutritional status, 27.4% of patients who were malnourished at baseline improved at 90 days, whereas the improvement rate was 52.7% among those at risk of malnutrition. Baseline characteristics and nutritional intervention variables according to nutritional improvement status are presented in Table 1. Age and baseline BMI were comparable between the two groups. Patients in the non-improvement group had a significantly higher CCI than those in the improvement group (2.62 ± 1.60 vs. 1.95 ± 1.28, *p* < 0.001). In contrast, patients in the improvement group had lower baseline MNA-SF scores (6.76 ± 2.40 vs. 8.17 ± 3.06, p < 0.001) and weaker handgrip strength (16.86 ± 8.43 kg vs. 19.01 ± 8.57 kg, *p* = 0.003) but higher daily energy intake at admission (980.53 ± 350.53 vs. 840.95 ± 332.16 kcal/day, *p* < 0.001). With respect to nutritional interventions, 181 (72.1%) patients in the improvement group received ONSs and 35 (13.9%) underwent enteral tube feeding during hospitalization; corresponding proportions in the non-improvement group were 67.4% (286/424) and 14.6% (62/424), respectively, with no significant between-group differences (all *p* > 0.05). During hospitalization, the improvement group achieved significantly higher rates of energy adequacy (80.9% vs. 59.4%, *p* < 0.001), protein adequacy (75.7% vs. 60.6%, *p* < 0.001), and adherence to nutritional intervention (78.9% vs. 68.6%, *p* = 0.004).

**Table 1:** Baseline characteristics and nutritional intervention during hospitalization according to nutritional improvement status at 90 days (*n* = 675).

Variables	Improvement group (*n* = 251)	Non-improvement group (*n* = 424)	*p* value
Demographic characteristics			
Age (years)	88.3 ± 4.7	88.5 ± 4.8	0.553
Men, *n* (%)	154 (61.4)	276 (65.1)	0.371
BMI (kg/m^2^)	20.9 ± 3.4	21.0 ± 3.1	0.700
Charlson Comorbidity Index	1.9 ± 1.2	2.6 ± 1.6	<0.001
Baseline nutritional and functional status			
MNA-SF score	6.8 ± 2.4	8.2 ± 3.1	<0.001
Barthel ADL score	47.5 ± 29.5	52.4 ± 30.5	0.066
Handgrip strength (kg)	16.9 ± 8.4	19.0 ± 8.5	0.003
Calf circumference (cm)	27.9 ± 5.4	28.3 ± 4.3	0.304
Nutritional intake at admission			
Daily energy intake (kcal/day)	980.5 ± 350.5	840.9 ± 332.1	<0.001
Daily protein intake (g/day)	54.3 ± 21.2	53.1 ± 21.4	0.452
Nutritional intervention during hospitalization			
Energy adequacy, *n* (%)	203 (80.9)	252 (59.4)	<0.001
Protein adequacy, *n* (%)	190 (75.7)	257 (60.6)	<0.001
Adherence to intervention, *n* (%)	198 (78.9)	291 (68.6)	0.004

Data are presented as the mean ± standard deviation or *n* (%).

Nutritional improvement is defined as a transition from “malnourished” to “at risk” or from “at risk” to “normal” based on MNA-SF classification at 90 days.

Energy adequacy is defined as achieving the estimated daily energy requirement during hospitalization.

Protein adequacy is defined as achieving the estimated daily protein requirement during hospitalization.

Adherence to nutritional intervention was defined as continued use of oral nutritional supplements at 90-day follow-up.

ADL, activities of daily living; BMI, body mass index; CCI, Charlson Comorbidity Index; MNA-SF, Mini Nutritional Assessment–Short Form.

In multivariable logistic regression analysis adjusting for age, baseline BMI, CCI, and baseline ADL score, a higher CCI was independently associated with a lower likelihood of nutritional improvement (per unit increase; OR = 0.751, 95% CI: 0.665–0.849, *p* < 0.001). Achieving energy adequacy (vs. not achieving; OR = 2.424, 95% CI: 1.511–3.887, *p* < 0.001) and good adherence to nutritional interventions (vs. poor adherence; OR = 1.921, 95% CI: 1.303–2.834, *p* < 0.001) were independently associated with nutritional improvement, whereas protein adequacy (adequate vs. inadequate) was not significantly associated with improvement (OR = 1.148, 95% CI: 0.740–1.782, *p* = 0.538) (Table 2). A sensitivity analysis excluding patients with normal nutritional status at baseline (*n* = 64) yielded similar results, with no substantial changes in the associations observed. The VIF values for energy adequacy and protein adequacy were 1.69 and 1.57, respectively, and all variables had VIF values of < 2, indicating no evidence of significant multicollinearity.

**Table 2:** Multivariable logistic regression analysis of factors associated with nutritional improvement at 90 days (*n* = 675).

Variables	Β (SE)	OR (95% CI)	*p* value
Age (per year increase)	0.007 (0.018)	1.007 (0.971–1.044)	0.669
Baseline BMI (per kg/m^2^ increase)	0.040 (0.026)	1.041 (0.988–1.096)	0.132
Charlson Comorbidity Index (per unit increase)	−0.286 (0.062)	0.751 (0.665–0.849)	<0.001
Baseline ADL score (per point increase)	−0.001 (0.003)	0.998 (0.993–1.002)	0.765
Energy adequacy (yes vs. no)	0.885 (0.241)	2.424 (1.511–3.887)	<0.001
Protein adequacy (yes vs. no)	0.138 (0.224)	1.148 (0.740–1.782)	0.538
Adherence to nutritional intervention (good vs. poor)	0.653 (0.198)	1.921 (1.303–2.834)	<0.001

Multivariable logistic regression model is adjusted for age, baseline BMI, Charlson Comorbidity Index, and baseline ADL score.

ADL, activities of daily living; BMI, body mass index; CI, confidence interval; OR, odds ratio; SE, standard error.

**Figure 2:**
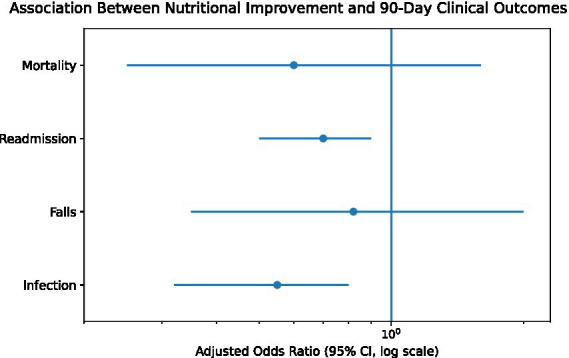
Adjusted associations between nutritional improvement and 90-day clinical outcomes. Adjusted odds ratios (ORs) and 95% confidence intervals (CIs) are estimated using multivariable logistic regression models adjusted for age, body mass index (BMI), Charlson Comorbidity Index, and baseline activities of daily living (ADL) score. The vertical reference line indicates an OR of 1.0. Values of < 1.0 indicate a lower risk of the outcome among patients who achieved nutritional improvement.

At 90 days, the improvement group demonstrated lower rates of mortality (1.2% vs. 2.8%), readmission (13.5% vs. 22.9%), falls (2.0% vs. 3.3%), and new infections (7.6% vs. 16.5%) compared with the non-improvement group. In unadjusted analyses, nutritional improvement (improvement vs. non-improvement) was significantly associated with reduced risks of readmission (OR = 0.528, 95% CI: 0.345–0.809, *p* = 0.003) and infection (OR = 0.414, 95% CI: 0.243–0.706, *p* < 0.001). After adjustment for age, sex, BMI, CCI, and baseline ADL score, these associations remained significant (readmission: OR = 0.545, 95% CI: 0.351–0.847, *p* = 0.007; infection: OR = 0.388, 95% CI: 0.225–0.671, *p* < 0.001). No significant associations were observed for mortality (adjusted OR = 0.433, 95% CI: 0.119–1.578, *p* = 0.205) or falls (adjusted OR = 0.685, 95% CI: 0.236–1.986, *p* = 0.486) (Table 3; Figure 2).

**Table 3:** Association between nutritional improvement (vs. non-improvement) and 90-day clinical outcomes.

Outcomes	Unadjusted OR (95% CI)	*p* value	Adjusted OR (95% CI)	*p* value
Mortality	0.415 (0.116–1.489)	0.177	0.433 (0.119–1.578)	0.205
Readmission	0.528 (0.345–0.809)	0.003	0.545 (0.351–0.847)	0.007
Falls	0.595 (0.212–1.673)	0.325	0.685 (0.236–1.986)	0.486
Infection	0.414 (0.243–0.706)	<0.001	0.388 (0.225–0.671)	<0.001

Adjusted for age, baseline BMI, Charlson Comorbidity Index, and baseline ADL score.

ADL, activities of daily living; BMI, body mass index; CI, confidence interval; OR, odds ratio.

## 4 Discussion

In this multicenter cohort study of hospitalized patients aged ≥ 80 years at nutritional risk, approximately one-third achieved improvement in nutritional status at 90 days. Notably, achieving daily energy intake targets and maintaining good adherence to nutritional interventions were independently associated with nutritional improvement. Furthermore, nutritional improvement was associated with reduced risks of readmission and new infection within 90 days. These findings highlight the association of adequate energy intake and sustained adherence to nutritional strategies with clinically meaningful nutritional recovery in oldest-old hospitalized patients.

Our findings underscore the association between adequate energy intake and nutritional recovery. Although both energy and protein adequacy were higher in the improvement group, only energy adequacy remained independently associated with nutritional improvement in multivariable analysis. Current international guidelines, including those from the ESPEN, emphasize the importance of adequate energy and protein provision in hospitalized older adults (9). In addition, the PROT-AGE study recommends higher protein intake for older adults, particularly in the context of acute illness, to support muscle maintenance and recovery (19). However, in our study, protein adequacy was not independently associated with nutritional improvement after multivariable adjustment, which may reflect the overriding importance of sufficient energy intake for effective protein utilization. Real-world observational studies suggest that energy deficits are particularly common among acutely ill and frail older individuals and may occur even before protein insufficiency becomes evident (20). In oldest-old patients with multimorbidity and inflammatory stress, inadequate caloric intake may result in persistent negative energy balance, causing dietary protein to be preferentially oxidized for energy metabolism rather than utilized for anabolic processes such as muscle protein synthesis and tissue repair (21). Therefore, adequate energy provision may represent a physiological prerequisite for effective protein utilization in this highly vulnerable population. This interpretation may explain why energy adequacy remained independently associated with nutritional improvement, whereas protein adequacy did not retain significant association after adjustment.

Baseline differences between groups should be considered when interpreting these findings. Patients in the improvement group had lower baseline MNA-SF scores and weaker handgrip strength at admission, indicating poorer nutritional and functional status, yet paradoxically exhibited higher energy intake. Because nutritional improvement was defined by transitions in MNA-SF categories, patients with lower initial scores may have had greater potential for improvement during follow-up, raising the possibility of regression to the mean. At the same time, despite poorer baseline nutritional and functional status, the relatively higher baseline energy intake observed in the improvement group may reflect partially preserved oral intake capacity, which could have facilitated achievement of energy adequacy and subsequent nutritional recovery. Therefore, residual confounding, regression to the mean, and potential selection bias cannot be excluded and the observed associations should be interpreted with caution.

Furthermore, our results highlight the importance of adherence to nutritional interventions in achieving meaningful improvement in nutritional status. Although nutritional prescriptions are routinely implemented during hospitalization, substantial discrepancies between prescribed and actual intake have been widely reported (22). In older adults, adherence is frequently compromised by geriatric syndromes such as anorexia of aging, dysphagia, polypharmacy, and cognitive impairment, all of which may reduce effective nutrient intake despite appropriate dietary planning (23, 24). Evidence from randomized controlled trials indicates that structured, individualized nutritional support combined with systematic monitoring can significantly improve clinical outcomes, including mortality and functional status, compared with usual care (25). Moreover, studies focusing on post-discharge care have shown that sustained nutritional intervention beyond hospitalization is often necessary to translate dietary prescriptions into measurable functional recovery and reduced complications (26). Consistent with these observations, current ESPEN guidelines advocate for structured nutritional care pathways that incorporate regular reassessment and follow-up to ensure effective implementation and continuity of care (9). Collectively, our findings suggest that effective implementation, monitoring, and adherence may be important factors associated with the success of nutritional strategies across care transitions (27).

Notably, nutritional improvement was independently associated with lower risks of readmission and new infection. These findings are clinically meaningful because hospitalized oldest-old patients often experience extensive healthcare use and substantial vulnerability to infectious complications. Our findings are also consistent with previous evidence linking malnutrition to impaired immune function, delayed wound healing, and increased healthcare utilization (28, 29). From a clinical perspective, the observed difference in readmission risk (13.5% vs. 22.9%) corresponds to an estimated number needed to treat of approximately 11, suggesting that nutritional recovery may have practical implications for reducing short-term healthcare burden in this vulnerable population. Similarly, the lower risk of infection observed in patients with nutritional improvement may reflect the important role of adequate nutritional status in supporting immune function and recovery from acute illness. In contrast, no significant associations were observed for mortality or falls. The lack of association with mortality may be partly explained by the relatively low number of death events within the 90-day follow-up period, which may have limited statistical power to detect moderate associations. In addition, mortality in oldest-old hospitalized patients depends strongly on multiple factors beyond nutritional status alone, including disease severity, frailty, and acute medical complications. This finding differs from that of the EFFORT trial, which demonstrated a mortality benefit of individualized nutritional support (8), possibly reflecting differences in study design, intervention intensity, and patient characteristics. Similarly, falls are multifactorial geriatric events influenced by neuromuscular function, cognition, balance impairment, medication use, and environmental factors (30), which may attenuate the independent contribution of nutritional recovery. While most prior studies have assessed nutritional status at a single time point, our results suggest that dynamic improvements in nutritional status may confer short-term clinical benefits.

This study has several notable strengths. First, it included a large and geographically diverse sample of oldest-old, hospitalized patients from 28 tertiary hospitals, enhancing the representativeness of the findings within the Chinese healthcare context. Second, unlike many previous studies that broadly include individuals aged ≥ 65 years, this analysis specifically focused on patients aged ≥ 80 years, a population with particularly high vulnerability to malnutrition and adverse outcomes. Third, by evaluating dynamic changes in MNA-SF grade rather than relying solely on baseline nutritional status, this study provides novel insight into the clinical significance of nutritional recovery during and after hospitalization.

Some limitations should also be considered. First, this was an observational cohort study. Although we adjusted for major demographic and clinical variables, residual confounding cannot be excluded. Unmeasured factors such as frailty, sarcopenia, cognitive impairment, disease severity, inflammatory burden, functional dependence, and social support may have influenced both nutritional recovery and clinical outcomes. Second, nutritional intake was estimated using dietary records, which may be subject to reporting inaccuracies and may not fully capture day-to-day variations in energy and protein consumption. Third, because this was a multicenter real-world study, nutritional interventions were not fully standardized across participating centers. Differences in clinical practice patterns, nutritional care resources, and implementation of nutritional support may have introduced treatment heterogeneity. In addition, hospital-level clustering was not explicitly accounted for in the regression analyses, and unmeasured center-level factors may have affected the estimated associations. Therefore, the findings should be interpreted as patient-level associations rather than as effects of a fully standardized nutritional intervention protocol.

## 5 Conclusion

Among hospitalized patients aged ≥ 80 years at nutritional risk, adequate energy intake and adherence to nutritional interventions were independently associated with nutritional improvement. Nutritional improvement was further associated with reduced risks of readmission and infection, although no significant associations were observed for mortality or falls. These findings highlight the potential clinical relevance of monitoring dynamic changes in nutritional status and support the implementation of structured nutritional care strategies in oldest-old, hospitalized populations.

## Data Availability

The raw data supporting the conclusions of this article will be made available by the authors, without undue reservation.

## References

[1] Salari N Darvishi N Bartina Y Keshavarzi F Hosseinian-Far M Mohammadi M. Global prevalence of malnutrition in older adults: a comprehensive systematic review and meta-analysis. Public Health Pract (Oxf). (2025) 9:100583. doi: 10.1016/j.puhip.2025.100583, 39885903 PMC11780955

[2] Botero L Banks MD Gordon EH Bauer J Young AM. Incidence and outcomes of in-hospital nutritional decline: a prospective observational cohort study in adult patients. Clin Nutr. (2024) 43:1057–64. doi: 10.1016/j.clnu.2024.03.014, 38569329

[3] Clotet-Vidal S Saez Prieto ME Duch Llorach P Gutiérrez ÁS Casademont Pou J Torres Bonafonte OH. Malnutrition, functional decline, and institutionalization in older adults after hospital discharge following community-acquired pneumonia. Nutrients. (2023) 16:11. doi: 10.3390/nu16010011, 38201841 PMC10780721

[4] Qing H Zhang XD Yang E Li HX Wei YL Chen W . Nutritional status and nutritional intervention of older inpatients in China. J Nutr Health Aging. (2024) 28:100169. doi: 10.1016/j.jnha.2024.100169, 38308922 PMC12880569

[5] Kaiser MJ Bauer JM Ramsch C Uter W Guigoz Y Cederholm T . Validation of the Mini nutritional assessment short-form (MNA-SF): a practical tool for identification of nutritional status. J Nutr Health Aging. (2009) 13:782–8. doi: 10.1007/s12603-009-0214-7, 19812868 PMC12878690

[6] Becker L Volkert D Christian Sieber C Gaßmann KG Ritt M. Predictability of a modified Mini- nutritional-assessment version on six-month and one-year mortality in hospitalized geriatric patients: a comparative analysis. Sci Rep. (2019) 9:9064. doi: 10.1038/s41598-019-45452-0, 31227778 PMC6588546

[7] Wei K Nyunt MSZ Gao Q Wee SL Yap KB Ng TP. Association of frailty and malnutrition with long-term functional and mortality outcomes among community-dwelling older adults: results from the Singapore longitudinal aging study 1. JAMA Netw Open. (2018) 1:e180650. doi: 10.1001/jamanetworkopen.2018.0650, 30646023 PMC6324309

[8] Schuetz P Fehr R Baechli V Geiser M Deiss M Gomes F . Individualised nutritional support in medical inpatients at nutritional risk: a randomised clinical trial. Lancet. (2019) 393:2312–21. doi: 10.1016/S0140-6736(18)32776-4, 31030981

[9] Volkert D Beck AM Cederholm T Cruz-Jentoft A Goisser S Hooper L . ESPEN guideline on clinical nutrition and hydration in geriatrics. Clin Nutr. (2019) 38:10–47. doi: 10.1016/j.clnu.2018.05.024, 30005900

[10] Morris S Cater JD Green MA Johnstone AM Brunstrom JM Stevenson EJ . Inadequacy of protein intake in older UK adults. Geriatrics (Basel). (2020) 5:6. doi: 10.3390/geriatrics5010006, 32059533 PMC7151458

[11] Dupertuis YM Kossovsky MP Kyle UG Raguso CA Genton L Pichard C. Food intake in 1707 hospitalised patients: a prospective comprehensive hospital survey. Clin Nutr. (2003) 22:115–23. doi: 10.1054/clnu.2002.0623, 12706127

[12] Ginzburg Y Shmilovitz I Monastyrsky N Endevelt R Shahar DR. Barriers for nutritional care in the transition from hospital to the community among older patients. Clin Nutr ESPEN. (2018) 25:56–62. doi: 10.1016/j.clnesp.2018.04.004, 29779819

[13] Jiang S Chen X Ma L Guo Q Luo L Wang Y . Evaluating the effect of nutritional support in geriatric inpatients classified by the GLIM criteria. J Nutr Health Aging. (2025) 29:100585. doi: 10.1016/j.jnha.2025.100585, 40412298 PMC12172969

[14] Kondrup J Allison SP Elia M Vellas B Plauth M Educational and Clinical Practice Committee, European Society of Parenteral and Enteral Nutrition (ESPEN) . ESPEN guidelines for nutrition screening 2002. Clin Nutr. (2003) 22:415–21. doi: 10.1016/S0261-5614(03)00098-0 12880610

[15] Gomes F Schuetz P Bounoure L Austin P Ballesteros-Pomar M Cederholm T . ESPEN guidelines on nutritional support for polymorbid internal medicine patients. Clin Nutr. (2018) 37:336–53. doi: 10.1016/j.clnu.2017.06.025, 28802519

[16] Rattray M Desbrow B Roberts S. Comparing nutritional requirements, provision and intakes among patients prescribed therapeutic diets in hospital: an observational study. Forum Nutr. (2017) 33:199–204. doi: 10.1016/j.nut.2016.07.015 28606570

[17] Charlson ME Pompei P Ales KL MacKenzie CR. A new method of classifying prognostic comorbidity in longitudinal studies: development and validation. J Chronic Dis. (1987) 40:373–83. doi: 10.1016/0021-9681(87)90171-8, 3558716

[18] Mahoney FI Barthel DW. Functional evaluation: the Barthel index. Md State Med J. (1965) 14:61–5. 14258950

[19] Bauer J Biolo G Cederholm T Cesari M Cruz-Jentoft AJ Morley JE . Evidence-based recommendations for optimal dietary protein intake in older people: a position paper from the PROT-AGE study group. J Am Med Dir Assoc. (2013) 14:542–59. doi: 10.1016/j.jamda.2013.05.021, 23867520

[20] Vest AR DiDomenico RJ Lichtenstein L Slater T Ekpo E Damluji AA . Malnutrition and cachexia in inpatients with acute cardiac conditions: a scientific statement from the American Heart Association. Circulation. (2026) 153:e1078–105. doi: 10.1161/CIR.0000000000001405, 41732869 PMC13250749

[21] Deutz NEP Bauer JM Barazzoni R Biolo G Boirie Y Bosy-Westphal A . Protein intake and exercise for optimal muscle function with aging: recommendations from the ESPEN expert group. Clin Nutr. (2014) 33:929–36. doi: 10.1016/j.clnu.2014.04.007, 24814383 PMC4208946

[22] Schindler K Pernicka E Laviano A Howard P Schütz T Bauer P . How nutritional risk is assessed and managed in European hospitals: a survey of 21,007 patients findings from the 2007–2008 cross-sectional nutritionDay survey. Clin Nutr. (2010) 29:552–9. doi: 10.1016/j.clnu.2010.04.001, 20434820

[23] Morley JE. Anorexia of aging: a true geriatric syndrome. J Nutr Health Aging. (2012) 16:422–5. doi: 10.1007/s12603-012-0061-9, 22555783 PMC12878074

[24] de Sire A Ferrillo M Lippi L Agostini F de Sire R Ferrara PE . Sarcopenic dysphagia, malnutrition, and oral frailty in elderly: a comprehensive review. Nutrients. (2022) 14:982. doi: 10.3390/nu14050982, 35267957 PMC8912303

[25] Kaegi-Braun N Faessli M Kilchoer F Dragusha S Tribolet P Gomes F . Nutritional trials using high protein strategies and long duration of support show strongest clinical effects on mortality: results of an updated systematic review and meta-analysis. Clin Nutr ESPEN. (2021) 45:45–54. doi: 10.1016/j.clnesp.2021.08.003, 34620354

[26] Neelemaat F Bosmans JE Thijs A Seidell JC van Bokhorst-de van der Schueren MAE. Oral nutritional support in malnourished elderly decreases functional limitations with no extra costs. Clin Nutr. (2012) 31:183–90. doi: 10.1016/j.clnu.2011.10.009 22071290

[27] Cawood AL Elia M Stratton RJ. Systematic review and meta-analysis of the effects of high protein oral nutritional supplements. Ageing Res Rev. (2012) 11:278–96. doi: 10.1016/j.arr.2011.12.008, 22212388

[28] Ghaly P Iliopoulos J Ahmad M. The role of nutrition in wound healing: An overview. Br J Nurs. (2021) 30:S38–42. doi: 10.12968/bjon.2021.30.5.S38, 33733851

[29] Correia MITD Waitzberg DL. The impact of malnutrition on morbidity, mortality, length of hospital stay and costs evaluated through a multivariate model analysis. Clin Nutr. (2003) 22:235–9. doi: 10.1016/S0261-5614(02)00215-7, 12765661

[30] Ambrose AF Paul G Hausdorff JM. Risk factors for falls among older adults: a review of the literature. Maturitas. (2013) 75:51–61. doi: 10.1016/j.maturitas.2013.02.009, 23523272

