# Supplementary material for: Factors associated with nutritional status improvement in hospitalized oldest-old adults at nutritional risk
Source: Front Nutr. 2026 Jul 20;13:1856364. doi: 10.3389/fnut.2026.1856364 (PMC13429457; doi:10.3389/fnut.2026.1856364)
Supplement: Supplementary file 1 [file Table_1.DOCX]

**Supplementary Table S1. Comparison of baseline characteristics between patients included in the final analysis and those lost to follow-up**

| **Variables** | **Included in final analysis**  **(n = 675)** | **Lost to follow-up**  **(n = 62)** | **P value** |
| --- | --- | --- | --- |
| **Demographic characteristics** |  |  |  |
| Age (years) | 88.5 ± 4.8 | 89.4 ± 5.0 | 0.143 |
| Men, n (%) | 430 (63.7) | 40 (64.5) | 0.749 |
| BMI (kg/m²) | 20.9 ± 3.3 | 20.5 ± 3.1 | 0.381 |
| Charlson Comorbidity Index | 2.4 ± 1.5 | 2.6 ± 1.5 | 0.198 |
| **Baseline nutritional and functional status** |  |  |  |
| MNA-SF score | 7.6 ± 2.9 | 7.7 ± 2.5 | 0.767 |
| Barthel ADL score | 51.6 ± 29.4 | 55.1 ± 25.5 | 0.409 |
| Handgrip strength (kg) | 18.2 ± 8.5 | 17.4 ± 6.9 | 0.500 |
| Calf circumference (cm) | 28.2 ± 4.7 | 27.8 ± 3.6 | 0.554 |
| **Nutritional intake at admission** |  |  |  |
| Daily energy intake (kcal/day) | 905.4 ± 375.2 | 960.7 ± 280.5 | 0.259 |
| Daily protein intake (g/day) | 54.5 ± 24.2 | 52.5 ± 18.1 | 0.356 |

Data are presented as the mean ± standard deviation or n (%).

Abbreviations: ADL, activities of daily living; BMI, body mass index; MNA-SF, Mini Nutritional Assessment–Short Form.
